# Video-based educational intervention associated with improved stroke literacy, self-efficacy, and patient satisfaction

**DOI:** 10.1371/journal.pone.0171952

**Published:** 2017-03-23

**Authors:** Mary Carter Denny, Farhaan Vahidy, Kim Y. T. Vu, Anjail Z. Sharrief, Sean I. Savitz

**Affiliations:** 1 Department of Neurology, Stroke Program, UTHealth, Houston, Texas, United States of America; 2 Department of Neurology, MedStar Georgetown University Hospital, Washington, DC, United States of America; 3 Memorial Hermann Hospital—Texas Medical Center, Houston, Texas, United States of America; Massachusetts General Hospital, UNITED STATES

## Abstract

**Background and purpose:**

Interventions are needed to improve stroke literacy among recent stroke survivors. We developed an educational video for patients hospitalized with acute ischemic stroke (AIS) and intracerebral hemorrhage (ICH).

**Methods:**

A 5-minute stroke education video was shown to our AIS and ICH patients admitted from March to June 2015. Demographics and a 5-minute protocol Montreal Cognitive Assessment were also collected. Questions related to stroke knowledge, self-efficacy, and patient satisfaction were answered before, immediately after, and 30 days after the video.

**Results:**

Among 250 screened, 102 patients consented, and 93 completed the video intervention. There was a significant difference between pre-video median knowledge score of 6 (IQR 4–7) and the post-video score of 7 (IQR 6–8; p<0.001) and between pre-video and the 30 day score of 7 (IQR 5–8; p = 0.04). There was a significant difference between the proportion of patients who were very certain in recognizing symptoms of a stroke pre- and post-video, which was maintained at 30-days (35.5% vs. 53.5%, p = 0.01; 35.5% vs. 54.4%, p = 0.02). The proportion who were “very satisfied” with their education post-video (74.2%) was significantly higher than pre-video (49.5%, p<0.01), and this was maintained at 30 days (75.4%, p<0.01). There was no association between MoCA scores and stroke knowledge acquisition or retention. There was no association between stroke knowledge acquisition and rates of home blood pressure monitoring or primary care provider follow-up.

**Conclusions:**

An educational video was associated with improved stroke knowledge, self-efficacy in recognizing stroke symptoms, and satisfaction with education in hospitalized stroke patients, which was maintained at 30 days after discharge.

## Introduction

Of the 795,000 strokes that occur in the United States annually, 185,000 are recurrent events.[1] Recurrent strokes are associated with increased morbidity and mortality as compared to first-time strokes.[2] Stroke survivors are more likely to have unrecognized vascular risk factors, including hypertension and diabetes, than those who have not suffered a stroke in the past.[3] However, up to 80% of vascular events after stroke may be prevented by modifying vascular risk factors through medical and behavioral interventions.[4] Even after suffering a stroke, patients’ knowledge of stroke symptoms and risk factors remain limited.[5,6] Therefore, stroke survivors are an important population to target for educational interventions.

Stroke literacy, previously defined as knowledge of stroke symptoms and stroke risk factors, is an important component of reducing the risk of recurrent stroke.[7] Recent stroke survivors and their caregivers often report unmet educational needs in all aspects of stroke care including causes of stroke, stroke prevention and stroke recovery.[8,9] Although improved stroke literacy alone is not sufficient, it is an important component in the approach to decrease secondary stroke risk. Moreover, providing appropriate stroke education to stroke survivors may be associated with improved patient satisfaction and a decreased risk of depression.[10] The Joint Commission and all major quality improvement organizations have recognized the importance of improving knowledge and therefore require stroke education be provided to hospitalized stroke patients and their families.[11] However, there is no broadly accepted best practice and few educational interventions have been specifically developed, implemented, and evaluated in the inpatient setting. The small number of stroke literacy studies that have been performed in recent stroke survivors have either utilized in-person written materials,[12–15] a computer program,[16] or in-person teaching sessions,[17] with variable and inconsistent impact on stroke knowledge and patient satisfaction.

Video-based educational interventions have been utilized for other chronic diseases in order to increase knowledge and promote health behavior change. Educational videos have been shown to be more effective than written materials at increasing knowledge and modifying health behaviors including cancer screening, heart failure self-care adherence, and HIV testing.[18] Therefore, video-based educational materials may be an effective educational tool for improving stroke literacy in hospitalized stroke patients.

The purpose of this study was to develop and pilot a patient-centered educational video to improve stroke literacy among patients with acute ischemic stroke (AIS) and intracerebral hemorrhage (ICH) at the time of hospital discharge. The aims of our study were therefore to (1) assess the feasibility of implementing a video-based educational intervention for hospitalized stroke patients, (2) evaluate patients’ baseline stroke literacy, and (3) assess the impact of the video on patients’ stroke knowledge, self-efficacy in recognizing stroke symptoms, and satisfaction with their stroke education. The results of this pilot study are intended to aid in the design and implementation of a future randomized-controlled trial of a video-based stroke education intervention trial.

## Materials and methods

### Study design and setting

We conducted a prospective study using a pre- and post-test design to evaluate a video-based educational intervention for patients hospitalized with AIS and ICH within 24 hours of their planned discharge. All patients were admitted to the stroke service at Memorial Hermann Hospital—Texas Medical Center (MHH-TMC), which is a high-volume, comprehensive stroke center (CSC) located in Houston, Texas. Recruitment took place over a three-month enrollment period from March to June 2015.

### Participants

Patients ≥ 18 years, admitted to the CSC at MHH-TMC with a discharge diagnosis of AIS or ICH were eligible. The diagnosis of AIS or ICH was confirmed radiographically on brain imaging and on clinical evaluation by a stroke neurologist. Patients were excluded if they were unable to comprehend the consent process and did not have a legally authorized representative (LAR) available. Study personnel were responsible for determining if a patient could not comprehend the consent process due to decreased level of consciousness or language impairment. However, aphasia and cognitive impairment were not explicitly used as criteria for exclusion. Patients were also excluded if they were under hospice care or were non-English speakers. Patients with a discharge diagnosis of subarachnoid hemorrhage, transient ischemic attack or stroke mimic, and those enrolled in another ongoing stroke education study were excluded. Potentially eligible patients were identified by study personnel in daily multi-disciplinary discharge rounds. Written informed consent for study participation was obtained from the patient or LAR. The study and informed consent procedure were approved by the institutional review board (IRB) at the University of Texas Health Science Center at Houston, and by the MHH-TMC clinical research committee. The study participant was provided with a copy of the signed informed consent.

### Intervention

We developed a 5-minute stroke literacy video to be shown to recent stroke survivors at the time of their hospital discharge. The video was based on input from multiple stakeholders including the following: stroke survivors, caregivers, physicians, nurses, physical and occupational therapists, social workers, hospital administrators and members of the IRB. The video script was written at a 6^th^ grade reading level in order to accommodate a wide range of literacy levels. The video intentionally features a racially, ethnically and agedly diverse cast of participants, which reflects and aims to appeal to our multi-cultural patient population. A simple animation was incorporated to demonstrate acute ischemic stroke and hemorrhagic stroke. The stroke knowledge concepts addressed in the video included: definition of a stroke, stroke symptom recognition and action of calling 911, stroke risk factors, stroke rehabilitation, stroke prevention, and the importance of outpatient clinic follow-up. The educational video was shown to the study participants on a bedside laptop to allow for adjustable positioning. Although some caregivers were present at the time of the intervention, they were not included as study participants.

**Fig 1:**
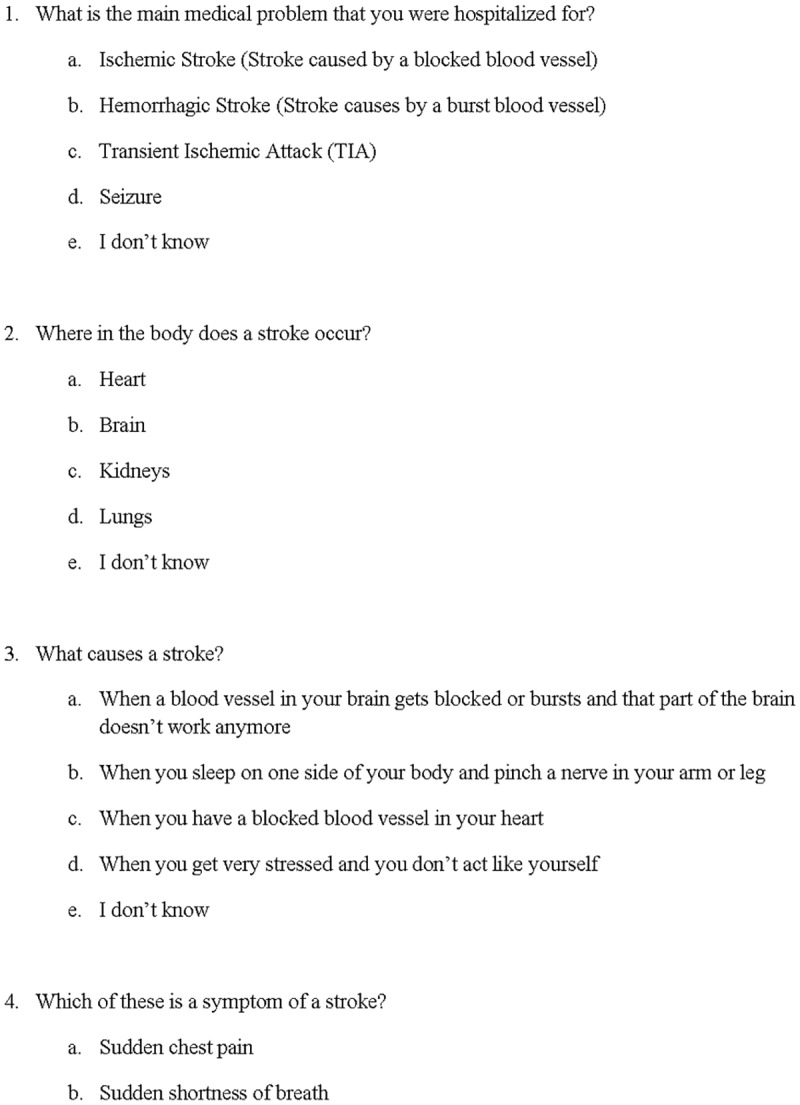
Stroke knowledge questionnaire.

We also developed a 10-item questionnaire, written at a 4^th^ grade reading level, to assess stroke knowledge, patient satisfaction with stroke education, and self-efficacy in recognizing stroke symptoms.[19,20] Items 1–8 assessed stroke knowledge using multiple choice format with five possible responses. There was only one correct response for each knowledge item. Items 9 and 10 assessed patients’ certainty in recognizing stroke symptoms (self-efficacy), and satisfaction with stroke education, respectively. Both items 9 and 10 were assessed using a 4-item Likert-type scales. The questionnaire, written at a 4^th^ grade reading level, was developed and edited by a multi-disciplinary team of stroke care providers, but has not been externally validated (Fig 1).

The questionnaire was administered to the stroke patients immediately before and after viewing the educational video. The patients were provided a copy of the questionnaire to read, but were also assisted in case they had difficulty reading. All patients independently responded to the questions; however the study staff circled the responses for patients with a dominant hemiparesis. The same questionnaire was also administered at 30-days after the intervention either by phone or in a clinic follow-up visit.

### Outcomes

We defined feasibility of implementing a video-based educational intervention for stroke patients as a success, if more than 80% of study population were able to complete all three facets of the intervention (pre- and post-video questionnaire and video viewing). In order to assess the impact of the video, we defined a Knowledge Score (KS) based on correct responses obtained on items 1–8 of the study questionnaire. The KS ranged from 0–8, with one point being awarded for each correct response obtained. No points were awarded for questions on which more than one response was provided or if the patient responded as “I don’t know”. Items 9 and 10 of the questionnaire were used to quantify self-efficacy in stroke symptom recognition and patient satisfaction with stroke education on a 4 point (0–3) Likert scale. Additional outcome assessments at 30-days included modified Rankin Scale (mRS) score, primary care provider (PCP) follow-up (yes/no) and home BP monitoring (yes/no), which were obtained from the patient’s caregiver in instances where the patient was unable to respond.

### Other measures and variables

We collected baseline demographic characteristics like age, gender, race/ethnicity, annual household income and educational level. Data on past medical history was later abstracted from medical records. All patients underwent the 5-minute protocol Montreal Cognitive Assessment (5-min MoCA) at admission and discharge for evaluation of their cognitive status.[21] Discharge mRS was also assessed. We also obtained feedback from study participants regarding their impressions of the video and suggestions for improvement.

### Sample size

The primary objectives of this study were (1) to assess the feasibility of administering a video-based stroke educational intervention prior to hospital discharge, and (2) to assess a change in stroke knowledge, self-efficacy, and satisfaction before and after the intervention. Based on our patient volumes, we estimated that 100 patients could be enrolled during a 3-month period, allowing us to satisfy the study objectives, as well as provide preliminary data to plan and implement a future randomized controlled trial.

### Statistical analyses

Descriptive analyses are used for reporting frequencies and proportions for categorical variables, and mean ± standard deviation or median with interquartile range for continuous variables. Chi square test and Fisher’s exact test was used to compare categorical baseline characteristics, whereas Wilcoxon rank-sum test was used for continuous variables. Various comparisons for the knowledge score were done using the Wilcoxon matched pair signed-rank test, and the comparison of proportions for self-efficacy and satisfaction items was done using McNemar test. Kolmogorov-Smirnov test was used to compare the pre- and post- video distribution of knowledge scores. Pearson correlation coefficient was determined for association between knowledge score and baseline 5-min MoCA. Alpha was set at 5% for all hypothesis testing. STATA Version 14 was used to conduct all analyses.[22]

## Results

**Fig 2:**
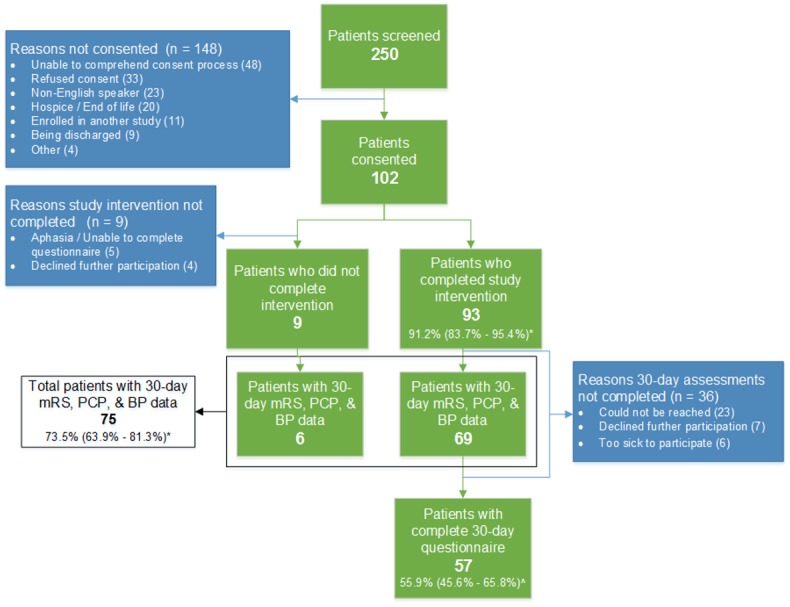
Flow diagram of study participants.

Feasibility metrics: Over a 3-month period from March–June 2015, we screened 250 patients of which 102 consented and 93 completed the intervention. This result translates into a 91.2% (95% CI: 83.7%–95.4%) success in administration of the video-based educational intervention. Of the patients completing the intervention, 75 (81.0%) had mRS, home blood pressure monitoring and PCP follow-up questions completed at 30 days, and 57 (61.3%) completed all 30-day assessments, including the repeat questionnaire. The most common reasons that patients did not enroll in the study were an inability to comprehend the consent process, refusal, or being a non-English speaker. The most common reasons for not completing 30-day assessments were inability to be contacted or declining further participation (Fig 2). Baseline characteristics of the patients who were consented, completed the intervention, and completed 30-day assessments are shown in Table 1. There were no clinically significant differences in the patient characteristics between those who completed the 30-day assessments as compared to those who did not (Table 1).

**Table 1:** Baseline characteristics.

	All consented patients (n = 102)	Patients completed study intervention (n = 93)	Patients completed study intervention (n = 93)		P value
			Patients with complete data (n = 57)	Patients with missing data (n = 36)	
Age, mean (SD)	62.9 (14.9)	62.1 (15.2)	60.2 (16.6)	65.1 (12.3)	0.11
Females, n(%)	33 (32.4)	30 (32.3)	20 (35.1)	10 (27.8)	0.46
Race/Ethnicity, n(%)					
• Caucasian	47 (46.1)	40 (43.0)	22 (38.6)	18 (50.0)	0.80
• African American	36 (35.3)	34 (36.6)	23 (40.4)	11 (30.6)	
• Hispanic/Latino	12 (11.8)	12 (12.9)	7 (12.3)	5 (13.9)	
• Asian	3 (1.9)	3 (3.2)	2 (3.5)	1 (2.8)	
• Other	4 (3.9)	4 (4.3)	3 (5.3)	1 (2.8)	
Ischemic Stroke, n (%)	65 (63.7)	57 (61.3)	36 (63.2)	21 (58.3)	0.89
Highest level of education, n(%)					
• Below high school	21 (20.6)	19 (20.4)	12 (21.1)	7 (19.4)	0.38
• High school	32 (31.4)	28 (30.1)	21 (36.8)	7 (19.4)	
• Some college	30 (29.4)	27 (29.0)	15 (26.3)	12 (33.3)	
• College degree	13 (12.8)	13 (13.9)	6 (10.5)	7 (19.4)	
• Professional degree	6 (5.9)	6 (6.5)	3 (5.3)	3 (8.3)	
Annual household Income, n(%)					
• Below $20,000	29 (28.4)	26 (27.9)	14 (24.6)	12 (34.3)	0.68
• $20,000–$34,999	25 (24.5)	24 (25.8)	15 (26.3)	9 (25.7)	
• $35,000–$74,999	22 (21.6)	19 (20.4)	13 (22.8)	6 (17.1)	
• $75,000 or more	17 (16.7)	17 (18.3)	10 (17.5)	7 (20.0)	
• Declined	7 (6.9)	6 (6.50)	5 (8.8)	1 (2.9)	
Baseline NIHSS, median (IQR)	5 (2, 10)	4 (2, 9)	4 (2, 10)	5 (2, 8)	0.97
Baseline 5-min MoCA, median (IQR)	8 (3, 10)	8 (5, 11)	8.5 (6, 11)	7 (2,10)	0.33
Discharge 5-min MoCA, median (IQR)	8 (6, 10)	9 (7, 10)	9 (7.5, 11)	8 (6, 10)	0.04
Hospital mRS, median (IQR)	3 (2, 4)	3 (2, 4)	3 (2, 4)	4 (3, 4)	0.09
Past medical history, n(%)					
• Dementia	4 (3.92)	3 (3.2)	1 (1.75)	2 (5.56)	0.31
• Hypertension	96 (94.1)	88 (94.6)	55 (96.5)	33 (91.7)	0.32
• Diabetes	37 (36.3)	33 (35.5)	17 (29.8)	16 (44.4)	0.15
• Hyperlipidemia	68 (66.7)	61 (65.6)	33 (57.9)	28 (77.8)	0.05
• Stroke	17 (16.7)	15 (16.1)	8 (14.0)	7 (19.4)	0.49

**Fig 3:**
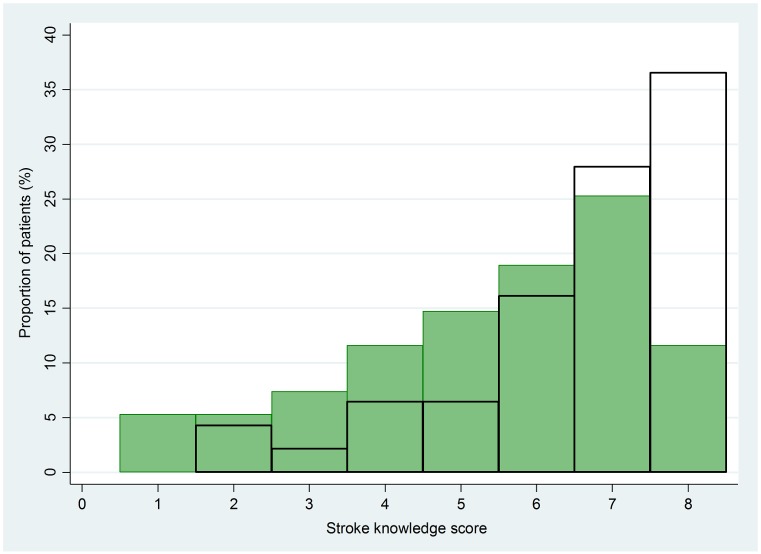
Shift in stroke knowledge scores. Distribution of stroke knowledge scores before the video (green bars) and after the video (black bars). The intervention resulted in a right shift of the distribution of knowledge scores which was statistically significant (p = 0.002 with Kolmogorov-Smirnov test).

Stroke Knowledge Score: We defined knowledge acquisition as a difference between pre- and post-video knowledge score (KS). We further defined knowledge retention as the difference between post-video and 30-day KS, whereas the net knowledge change was defined as the difference between pre-video and 30-day KS. There was a statistically signification increase in the pre- and post-video KS, median (Q1,Q3) [6(4–7) vs. 7(6–8)–p < 0.001]. At 30 days, the median (Q1,Q3) KS was 7 (5–8), which was not statistically different from the post-video KS. The 30-day KS however remained significantly higher than the pre-video KS (p = 0.04). These results are summarized in Table 2. The stroke video intervention resulted in an overall right shift in the distribution of KS among the patients who completed the intervention (Fig 3). There were no differences in pre, post, and 30-day knowledge scores with respect to age, gender, income, educational level, stroke severity, and 30 day mRS outcomes.

**Table 2:** Stroke knowledge scores.

Stroke knowledge score comparisons for different time points	Number of patients	Pre Score Median (IQR)	Post Score Median (IQR)	Day 30 Score Median (IQR)	P value
Pre vs. Post Intervention	93	6 (4, 7)	7 (6, 8)	-	< 0.001
Post vs. 30-Day after intervention	57	-	7 (6, 8)	7 (5, 8)	0.15
Pre vs. 30-Day after intervention	57	6 (5, 7)	-	7 (5, 8)	0.04

Self-efficacy in stroke symptom recognition: There was a significant difference between the proportion of patients who were “very certain” in recognizing stroke symptoms pre- and post-video (35.5% vs. 53.3% p = 0.01). The proportion of patients who continued to be “very certain” in stroke symptom recognition at 30-day assessment was also significantly higher than the pre-video time point (35.5% vs. 54.4%, p = 0.02). There was no difference between the proportion of patients who were “very certain” post-video and at 30 days (53.3% vs. 54.4%, p = 0.89).

Satisfaction with stroke education: Before the video, 49.5% of the participants were “very satisfied” with the stroke education they had received in the hospital, whereas after the video this proportion increased to 74.2% (p < 0.01). There was also a significant difference in the proportion of “very satisfied” patients between pre-video and at 30 days (49.5% vs. 75.4%, p < 0.01). There was no difference between the proportion of patients who were “very satisfied” post-video and at 30 days (74.2% vs. 75.4%, p = 0.87).

Cognitive Testing: Baseline 5-min MoCA scores were significantly correlated with pre-, post- and 30-day knowledge scores (r = 0.41, p<0.01; r = 0.47, p<0.01; r = 0.40, p < 0.01). However, there was no significant association between 5-min MoCA score and stroke knowledge acquisition or knowledge retention.

Other Outcomes: From among the patients who were assessed at 30 days for additional outcomes, 54.1% reported that they had a follow up visit with their primary care provider (PCP), and 78.4% reported checking their blood pressure at home. The median (Q1, Q3) mRS for these patients was 3 (1,4). We did not find any significant associations between these outcomes and KS.

## Discussion

Secondary stroke prevention requires new initiatives and approaches to better manage vascular co-morbidities and reduce the incidence of recurrent strokes. Stroke literacy is an important component of stroke prevention. Prior educational intervention studies using written information packets and in-person teaching session interventions or a multi-media computer program have reported variable results in improving stroke knowledge and limited success in improving patient satisfaction and perceived health status among stroke survivors.[23] Written materials, unfortunately, are often at too high a reading level and inadequate in meeting stroke survivors’ educational needs.[24]

We took a novel approach by implementing an educational video prior to hospital discharge in AIS and ICH patients. The use of a video as an educational tool offers the benefits of providing standardized content across learners, being less resource intensive than written materials or educational sessions and has been shown to be effective among viewers of low literacy levels for other medical conditions.[18] The choice of educational content for the video was based on The Joint Commission requirements and commonly asked questions posed in our stroke support group sessions of hospitalized patients. The video was designed in a question and answer format with a mock stroke patient asking questions and then obtaining answers to these questions from a multi-disciplinary and multi-cultural stroke care team including nurses, physicians, imaging technologists, speech therapists, physical therapists, occupational therapists and social workers. The video was intended to increase stroke knowledge and self-efficacy based upon the techniques of persuasive communication, imagery, and modeling, which have been shown to affect behavior change.[25] A 5-minute duration was chosen in order to deliver a limited number of simple messages and maintain attention. Prior studies had shown the efficacy of a stroke education video intervention in improving stroke knowledge among the public in an urban emergency department waiting area; however, this study did not specifically target patients with suspected stroke or stroke survivors.[26]

We found that implementing the educational video in the acute hospital setting was feasible. Over 90% of the consented patients completed the intervention. The main logistical challenges that we faced in showing the video was the distraction of providers entering and leaving the patients’ room and the ambient noises of the hospital. In future studies, the use of headphones for patients and caregivers would be helpful. In addition, we aimed to show the video to patients and available caregivers within 24 hours of their planned discharge; however their planned discharge time sometimes changed, which did lead to variability in when the patients viewed the video.

Our study population included both AIS and ICH patients of mild to moderate severity. The mean age of our study population was 62 years old with a range from 20 to 95 years old. Only one third of our participants were female. A broad range of annual household income levels and education levels were represented. The study population included participants from our racially and ethnically diverse patient population with 46% Caucasians, 37% African Americans and 13% Hispanics. Therefore, we believe that the video could be applicable to a range of patients hospitalized for mild to moderate acute ischemic stroke and intracerebral hemorrhage.

We found that the stroke education video was associated with improved stroke knowledge, certainty in recognizing stroke symptoms (self-efficacy), and satisfaction with stroke education immediately after viewing the video and at 30 days after hospitalization. Baseline stroke knowledge score were higher than we expected prior to conducting the study (median 6 out of 8). The high baseline KS scores may be due to the nature of the questionnaire and the additional education the patients received from the nursing and physician staff. The stroke video intervention served as an adjunct to the verbal and written stroke education that all patients are provided. While our study was not designed to assess for differences in specific patient populations, there were no clear patterns identifying which patient populations particularly benefited from the intervention or which populations did not show knowledge acquisition and retention. Interestingly, the 5-min MoCA cognitive screening tool was strongly correlated with stroke knowledge before, immediately after and 30 days after the educational intervention, but was not associated with the ability to acquire or retain stroke knowledge. While the primary objective of our study was knowledge acquisition, we did collect information on whether the patients made an appointment with their PCP and checked their blood pressure at home. Neither of these outcomes were found to be associated with knowledge retention but our sample number was likely too low to detect any potential associations.

Our study has the limitations of being single arm and non-randomized with substantial loss to follow-up. The overall 30-day follow-up rate was 81.0% with only 61.3% completing the 30-day questionnaire. Loss to follow-up was a particular challenge in this patient population as participants were often still in acute inpatient rehab or a skilled nursing facility, which made contacting them more difficult. However, the follow-up rate is comparable to a previously reported inpatient stroke education trial.[17] Another potential limitation is that it is unknown how the statistical median increase of 1 point on our questionnaire may affect behavioral changes to promote vascular risk factor control. Moreover, we did notcontrol for the variability in additional stroke education that participants may receive after they are discharged from the acute care hospital prior to the 30-day follow-up assessments. In addition, our questionnaire has not undergone testing for reliability or validity. However, we believe involvement of a multi-disciplinary team enhanced the face validity of our questionnaire.

A randomized trial will clearly be needed to evaluate the efficacy of the video as a tool to improve stroke knowledge, self-efficacy and patient satisfaction. The findings of the study presented here will be incorporated into the design of our forthcoming randomized trial. Moreover, given the available evidence that repetition facilitates learning, the video will be shown to participants on multiple occasions with the opportunity to ask questions.[27] We will address the importance of building on knowledge acquisition in order to impact behavioral change and self-efficacy by incorporating, for example, a skill-building activity such as home blood pressure monitoring to promote a health behavior associated with stroke risk reduction. In addition, we plan to assess for population differences in ethnicity, gender, and age. Lastly, using a video may need to be compared against written materials or multimedia programs to assess the most effective approaches to promote stroke literacy in recent stroke survivors.
